# Identification of susceptibility loci for complex diseases in a case-control association study using the Genetic Analysis Workshop 14 dataset

**DOI:** 10.1186/1471-2156-6-S1-S102

**Published:** 2005-12-30

**Authors:** Kimberly F Kerstann, Kevin Jacobs, Xiaohong (Rose) Yang, Andrew W Bergen, Lynn R Goldin, Alisa M Goldstein

**Affiliations:** 1 Division of Cancer Epidemiology and Genetics, National Cancer Institute, NIH, DHHS, Bethesda, Maryland, USA; 2 BioInformed LLC, Cleveland, OH

## Abstract

Although current methods in genetic epidemiology have been extremely successful in identifying genetic loci responsible for Mendelian traits, most common diseases do not follow simple Mendelian modes of inheritance. It is important to consider how our current methodologies function in the realm of complex diseases. The aim of this study was to determine the ability of conventional association methods to fine map a locus of interest. Six study populations were selected from 10 replicates (New York) from the Genetic Analysis Workshop 14 simulated dataset and analyzed for association between the disease trait and locus D2. Genotypes from 45 single-nucleotide polymorphisms in the telomeric region of chromosome 3 were analyzed by Pearson's chi-square tests for independence to test for association with the disease trait of interest. A significant association was detected within the region; however, it was found 3 cM from the documented location of the D2 disease locus. This result was most likely due to the method used for data simulation. In general, this study showed that conventional case-control association methods could detect disease loci responsible for the development of complex traits.

## Background

Current linkage and association methods have proven to be extremely successful in identifying genetic loci responsible for the development of diseases shown to have simple Mendelian modes of inheritance. However, most inherited diseases do not follow simple Mendelian patterns. The etiologies of these diseases are considered to be complex in nature, consisting of the interaction of multiple genetic loci as well as possible environmental factors. Given the complex nature of these diseases, how do we adequately identify genetic loci responsible for their development? Are the current methods sufficient to tease out one of the possibly many loci responsible for the development of a complex trait?

The phenotype modelled in the simulated data set is a wonderful example of a complex trait. The behavioral disorder, Kofendrerd Personality Disorder (KPD), is characterized by the presence of any one of 12 clinical characteristics, which may be divided into three diagnostic groups (phenotype 1, 2, and 3). Each of these phenotypic groups is the result of simultaneous mutations in at least 2 loci with varying modes of inheritance. For our analysis we have selected to isolate locus D2 by association methods.

In a complimentary paper presented at the workshop, Yang et al. used linkage analysis to map the D2 locus to the telomeric region of chromosome 3, which is in keeping with the expected result [1]. Association studies have long been accepted as an appropriate follow-up study for linkage analyses to fine map the disease locus of interest. The goal of our analysis was to determine whether a case-control association study could precisely map the D2 locus.

## Methods

Given the goals of our study, we needed to know the location of the susceptibility locus of interest, D2. We therefore looked at the answers before analysis. The selected locus D2 was described as dominant with a disease allele frequency of 0.15. This locus was implicated in the development of 4 of the 12 clinical characteristics traits e, f, h, and k. To increase genetic homogeneity, and thus power to detect an association, we redefined the affection status so that individuals were classified as affected only if they possessed these four clinical characteristics.

### Marker selection

Disease locus D2 is located at the telomere of chromosome 3. We thus purchased 3 SNP sets (packets 152, 153, 154), which included the linkage peak observed froma 7-cM map of chromosome 3[1]. Each purchased marker set contained 20 markers that were on average 0.3 cM apart. After removing duplicate SNPs between marker sets we created an analysis set containing 45 SNPs (B03T3021 to B03T3067) from a 12-cMtelomeric region of chromosome 3. Genotype data from 10 replicates of the New York (NY) population were obtained. Genotype data were also obtained for 500 unrelated controls with no family history of KPD.

### Case-control selection

In order to maintain consistency within our group we analyzed the NY population; the extended pedigree structure was more appropriate for our initial linkage analysis [1]. Ten replicates from the NY dataset were selected. From this population, five study populations were randomly selected by the SAS *surveyselect *function, taking care that only one affected individual from a family was selected as a case. For each case population a control population consisting of twice the number of cases was randomly selected, also with the SAS *surveyselect *function. The details of each case population are as follows: populations 1–3 contained 50 cases each. These three replicates provided at least 80% power (estimated by QUANTO Version 0.5 [2]) to detect an association given a dominant model and a genetic effect of at least 3. These replicates provided a comparison to determine the degree of inter-study variability expected based on case selection alone. Populations 4–6 contained 250 cases each; this case size was selected to provide greater than 80% power to detect the simulated effect, given a dominant model and a genotype-specific relative risk of at least 3. Population 4 used the redefined and more homogeneous definition for affection status. In contrast, population 5 used the original definition of affection status provided in the dataset. Population 5 is therefore assumed to be more heterogeneous than population 4. Population 6 contained a case population selected by the MERLIN [3] 'select' function using the redefined affection status. This process allowed the selection of cases that represent the "best-linked" individual from a pedigree [4].

### Analysis

For all study populations 2 × 3 contingency tables were generated based on genotype counts in the case and control populations. Pearson's chi-square tests for independence were performed on the tables and *p*-values were compared between study populations. This analysis was considered to be the most general analysis for a case-control association study. Given that the description of the simulation model indicated the presence strong linkage disequilibrium (LD) in the region of interest, we assayed LD using the program HAPLOVIEW (*Haploview v2.05*). We calculated D' (normalized value for D calculated as D'_ij _= D_ij_/D_max_) for all pair-wise marker comparisons. We also used HAPLOVIEW to compare haplotype frequencies between the case and control populations.

## Results

**Figure 1:**
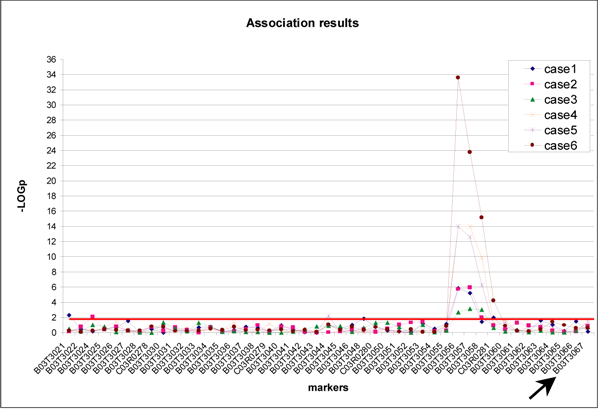
***p*-Values obtained from chi-square tests for independence, plotted as -log_10_(p-value)**. Value equivalent to *p *= 0.001 is indicated by bold red line. Case 1–6 reflects study populations selected as defined in the text. Case 1–3: 50 cases, 100 controls; Case 4: 250 cases, 500 controls; Case 5: 250 cases (original affection), 500 controls; Case 6: 250 cases (cases selected by MERLIN select function), 500 controls. Black arrow indicates marker documented to be linked to the disease locus.

Genotypes from all SNPs (B03T3021 to B03T3067) in a 12-cM region at the telomere of chromosome 3 were tested for association with the disease trait by chi-square test for independence. The results obtained from all populations are shown in Figure 1, plotted as the -log_10_(*p*-value). As expected from the number of tests performed, a *p*-value of 0.05 or even 0.01 was not stringent enough to identify a true significant association, as many false positives were observed. Therefore, results with a *p*-value of 0.001 or less were considered to be significant as determined by Bonferroni correction. A significant association was detected between markers B03T3056, B03T3057, B03T3058, and C03R0281 and the disease trait. This result was confirmed in population 5; the replicate selected using the original disease definition. However, tests with this case population produced lower significance levels. No association was detected for SNPs closer to the disease locus, which was reported to be linked to SNP B03T3067.

**Figure 2:**
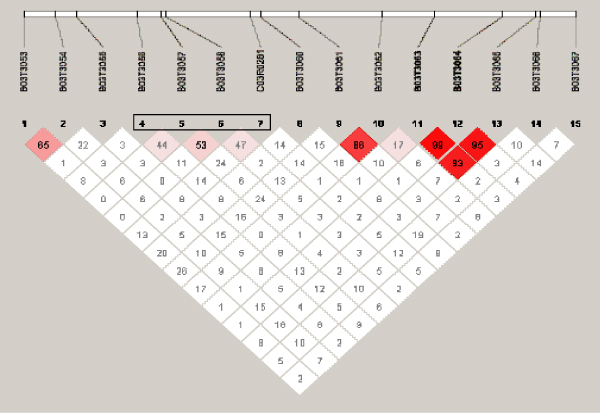
**Degree of linkage disequilibrium present within region of interest**. Output obtained from HAPLOVIEW assessing the degree of LD present within region. The four SNPs that showed the most significant association (B03T3056, B03T3057, B03T3058, and C03R0281) with the trait are outlined by a black rectangle. Each square represents a pair-wise comparison and the number within the square indicates the D' value. Significant LD is indicated by a red square.

The control population was used to assess the degree of LD present within a narrowed region of chromosome 3, from marker B03T3053 to B03T3067. This region was reported in the simulated dataset to contain significant LD. Figure 2 shows the patterns of LD present in this region. LD was found to be very sparse within this region and a large number of haplotypes with low frequencies were observed. Given these two factors we determined that haplotype analysis would offer little additional information over our initial analysis based on genotypes alone and therefore we did not pursue haplotype analysis further.

## Discussion

With the knowledge of the answers for the simulated dataset, we decided to isolate the disease locus D2 with standard methods, i.e., linkage analysis followed by association analysis. A complementary analysis presented from our group showed a significant linkage peak in the telomeric region of chromosome 3 [1]. This peak was observed within a region expected considering the physical location of locus D2. Our aim was to determine whether association studies could be used to fine map this disease locus of interest. Analysis of a 12-cM region near the telomere of chromosome 3 revealed a statistically significant association between genotypes at 4 markers (B03T3056, B03T3057, B03T3058, and C03R0281) and the clinical characteristics e, f, h, and k. These loci were significant after correction for multiple tests. One limitation of our study is that our smaller samples (*n *= 50) were slightly underpowered given the large number of loci tested. However, we were most interested in determining whether a linkage peak could be narrowed down using the association tests so we focused on the pattern of results across the region rather than the actual significance level.

Without prior knowledge of the disease locus one would feel that this study was highly successful. Linkage analysis identified a minimal disease region of approximately 3 cM linked to the disease trait [1]. We showed significant association with the disease trait and a narrow region (<1 cM) of chromosome 3, thus fine mapping a region associated with an increased risk of disease. However, the simulated disease model clearly stated that the disease locus D2 was linked to the last marker on the chromosome. This marker was nearly 3 cM from the peak of significant association. This finding was replicated using the original affection status, indicating that this result was not due to redefining the affection status.

Several groups using various analytical methods at Genetic Analysis Workshop 14 observed the same association peak. The association peak 3 cM from the actual disease locus most likely resulted from the methods used for the data simulation. For this reason our results and conclusions may not be directly applicable to real datasets. With this in mind we are able to make some general conclusions. Case selection can greatly influence both power and locus detection. As shown in the analysis in which MERLIN was used to select the 250 cases from linked families, power increased dramatically over that observed from 250 randomly selected cases. Power may be increased by refining the phenotype definition, although this was not necessary for locus detection in this particular example. As expected, an increase in the number of selected cases not only increased the significance of association, but also decreased the variability in observed significance. However, even the slightly under-powered replicates were able to detect the correct location of association. We were able to show that a case-control approach could detect an association in a linked region but because there was very little LD present in the simulated dataset, we could not determine how well this approach could fine map an actual disease locus.

## Abbreviations

LD: Linkage disequilibrium

KPD: Kofendrerd Personality Disorder

## Authors' contributions

KFK participated in study design, performed the statistical analyses, and drafted the manuscript. KJ performed data management and provided technical consulting. XY and AWB participated in study design, results interpretation, and aided in drafting the manuscript. LRG and AMG aided in the establishment of study goals, analysis direction, and preparation of manuscript. All authors have read and approved the final manuscript.
